# Differential impacts of ridesharing on alcohol-related crashes by socioeconomic municipalities: rate of technology adoption matters

**DOI:** 10.1186/s12889-021-12066-z

**Published:** 2021-11-04

**Authors:** Carola Blazquez, José Guillermo Cedeño Laurent, José Ignacio Nazif-Munoz

**Affiliations:** 1 Department of Engineering Sciences, Universidad Andres Bello, Quillota 980, Viña del Mar, Chile; 2 Department of Environmental Health, Harvard T.H. Chan School of Public Health, 677 Huntington Avenue, Boston, MA 02115 USA; 3 Université de Sherbrooke, Faculté de médecine et des sciences de la santé, 150, place Charles-Le Moyne, Longueuil, QC J4K 0A8 Canada

**Keywords:** Uber, Road safety, Alcohol, Socioeconomic groups

## Abstract

**Background:**

An emergent group of studies have examined the extent under which ridesharing may decrease alcohol-related crashes in countries such as United States, United Kingdom, Brazil, and Chile. Virtually all existent studies have assumed that ridesharing is equally distributed across socioeconomic groups, potentially masking differences across them. We contribute to this literature by studying how socioeconomic status at the municipal level impacts Uber’s effect on alcohol-related crashes.

**Methods:**

We use data provided by Chile’s Road Safety Commission considering all alcohol-related crashes, and fatal and severe alcohol-related injuries that occurred between January 2013 and September 2013 (before Uber) and January and September 2014 (with Uber) in Santiago. We first apply spatial autocorrelation techniques to examine the level of spatial dependence between the location of alcohol-related crashes with and without Uber. We then apply random-effects meta-analysis to obtain risk ratios of alcohol-related crashes by considering socioeconomic municipality differences before and after the introduction of Uber.

**Results:**

In both analyses, we find that the first 9 months of Uber in Santiago is associated with significant rate ratio decreases (RR = 0.71 [95% Confidence Interval (C.I.) 0.56, 0.89]) in high socioeconomic municipalities in all alcohol-related crashes and null (RR = 1.10 [95% C.I. 0.97, 1.23]) increases in low socioeconomic municipalities. No concomitant associations were observed in fatal alcohol-related crashes regardless of the socioeconomic municipality group.

**Conclusions:**

One interpretation for the decline in alcohol-related crashes in high socioeconomic municipalities is that Uber may be a substitute form of transport for those individuals who have access to credit cards, and thus, could afford to pay for this service at the time they have consumed alcohol. Slight increases of alcohol-related crashes in low socioeconomic municipalities should be studied further since this could be related to different phenomena such as increases in alcohol sales and consumption, less access to the provision of public transport services in these jurisdictions, or biases in police reports.

**Supplementary Information:**

The online version contains supplementary material available at 10.1186/s12889-021-12066-z.

## Background

Ridesharing, a service that connects drivers with potential passengers through a mobile application, is a type of technological innovation, which has shown a remarkable case of diffusion and convergence across and within transport systems. For instance, Uber, one of many ridesharing application providers, has since 2011 spread to at least 900 cities in 84 countries, and during 2018 it supplied over 10 billion trips globally [1]. In the Middle East, North Africa, and South Asia, Careem has spread to more than 50 cities [2]. In United States, Lyft operates in more than 300 cities, and in China, DiDi Chuxing is used by more than 450 million users, and spread to more than 400 cities [3].

The understanding of the multifaceted impacts of ridesharing however requires a theoretical understanding of how and why this type of services successfully diffuse within transport systems. The theory of diffusion innovations [4] has been used to explain the adoption of ideas and technologies in different realms including among others, science, policies, marketing, and transport [5, 6]. This theory describes how in a given community during a specific period, members are likely to adopt a similar device (convergence), which responds to a specific unmet need. Convergence however may be slow but also may not occur at all. Indeed, social networks, whereby technologies or ideas are channelled, can impede spreading. This may be because the innovation is not properly communicated or understood, does not match a need, or means to adopt it are not yet available [7, 8]. Considerable part of diffusion of innovation research has focused on classifying what makes individuals to adopt before convergence could be observed. Broadly, when an innovation has finally spread, five overlapping groups of users could be identified: innovators, early adopters, early majority, late adopters and laggards or sceptics [9].

The functioning of transport systems can be positively or negatively altered depending on how rapidly transport providers and/or users accept new technological innovations [10]. This has been the case for instance of mobile ticketing service in public transport [11], models of bike sharing [12], alternative fuel vehicles [13], seat belts [14] or child restraints [15]. Ultimately, the introduction of these technologies can be associated with less polluted environments after alternative fuel vehicles cope the market [16], or considerable decreases in traffic fatalities after seat belts or child restrains are both massively and properly used [17–19]. Yet, the adoption and further use of transport-related technologies may not be equally accepted by transport providers or users [20].

Regarding the diffusion of ridesharing applications two elements should be highlighted. First, studies have suggested that early adopters of ridesharing are both highly educated and from high-income households [21–25]. Further, the initial use of this service was associated with ease of payment since trips could be charged to credit cards, which were integrated into cell phone applications [22]. To extend its use, many ridesharing applications have been complemented with in-cash payments [26, 27]. Second, another group of studies have indicated that ridesharing services could be also associated with decreasing of congestion [28], decreasing in public transit use [29], reduction of air quality [30], and declining of alcohol-related crashes [31–36]. More specifically, in terms of alcohol-related crashes variation, it has been argued that ridesharing provides a valuable alternative to drinking and driving when access to public transport is limited (i.e., low frequency of provision during late hours) and/or traditional taxis costs may be unknown (i.e., high fluctuation of prices for similar trips).

Despite the interest in and growth of ridesharing studies and alcohol-related crashes, there is a surprising gap on understanding how changes in the diffusion of ridesharing use could be associated with variation of these outcomes. This is important because part of the literature has reported mixed results. Greenwood and Wattal [32], Peck [33], Morrison et al. [34], and Martin-Buck [37] have indicated reductions in alcohol-related crashes after ridesharing increased over time. Conversely, Brazil and Kirk [31, 38], Dills and Mulholland [39], and Nazif-Munoz et al. [36] did not observe any change in the occurrence of these crashes, even after exploring potential increments. The absence of significant variation in these former studies possibly mask either short terms associations or changes in specific groups—such as innovators and/or early adopters. However, to better understand ridesharing application associations, it should be acknowledged that the use of this technology follows over time a gradient of socioeconomic status (SES), whereby innovators and early adopters are more likely to belong to high-income groups, and late adopters and laggards may be linked with lower-income groups [21–25]. As such, when ridesharing applications begin emerging in an urban community, transport-related changes would be expected in high-income groups more prominently [40], and then, depending on whether convergence of this application is reached, outcomes at the population level, regardless of the socioeconomic gradient, should be observed.

In this work, we put lens on understanding how the early adoption of one ridesharing model—Uber—in Chile’s capital city, Santiago, could be associated with alcohol-related crash variations. Uber was introduced in January of 2014 in Santiago and by 2017, this service was active in 16 new Chilean cities. At that time, in Santiago, more than 35,000 individuals were registered as drivers, more than 1,350,000 individuals used the application per day, and initial reports suggested that this service was offered throughout all its municipalities [41]. The case of Santiago is interesting due to the following reasons. First, a previous study [36] has indeed suggested the absence of effects between Uber and alcohol-related traffic crashes in this city. However, this study did not consider adoption pattern differences when assessing the impact of this service. As a result, this study could have masked different effects across Uber users. Early adopters and frequent users of Uber in Chile, however, are from high-income groups [42]. Further, during the first 2 years of Uber’s implementation (2014 to 2016), this platform limited its use to credit card holders only [42], but credit cards are not equally distributed across income groups in Chile [43, 44]. Indeed, in this country, individuals at the highest income decile relative to individuals from the lowest decile, have approximately 50 more chances of owning a credit card [45]. This reinforces the notion that high-income individuals were more likely to be Uber users than individuals from lower income groups. Therefore, possible associations between this service, at initial stages, and alcohol-related crashes should consider socioeconomic differences more consistently. Second, in terms of alcohol consumption while no differences across socioeconomic groups are observed regarding heavy drinking and heavy episodic drinking, individuals from higher SES are more likely to drink higher volumes of pure alcohol weekly than any other socioeconomic group [46]. Lastly, Santiago is a highly socially segregated city, its east central sector, made up of seven municipalities, contains more than 80% of the main population of the richest quintile. Under these characteristics it could be assumed from an ecological perspective that ridesharing in its genesis could be associated with reductions in alcohol-related outcomes in municipalities where high-income individuals inhabit, and no associations would be necessarily observed in municipalities where individuals from lower incomes live.

To fill gaps in this literature, we examine how SES at the municipal level impacts Uber’s effect on alcohol-related crashes in Santiago, Chile by focusing on the slow pace of Uber’s implementation during its first 9 months of implementation. We hypothesize that, in municipalities where high-income individuals inhabit, the beginning of Uber operations relative to its absence, would be associated with lower risks of alcohol-involved crashes.

## Methods

### Data

The road traffic crash database was requested to the Chile’s National Commission of Road Safety (CONASET) through the Transparency Law [47]. Chilean police (*Carabineros de Chile*) collect and report the traffic crash data that consists of crash attributes (date, time, location, contributing cause, type of crash, etc.) and information of the involved victims (age, gender, road user type, injury severity, etc.). Police officers indicate whether alcohol was involved or played a role in the occurrence of the crash by detecting the presence of alcohol through the application of breathalysers [48]. The information provided by police officers is registered as “alcohol-related” when the road user involved in the crash has surpassed a blood alcohol concentration level of 0.03% [49]. We used alcohol-related traffic crashes that occurred in Santiago, Chile, in which the driver, passenger, or pedestrian was under the influence of alcohol at the moment of the crash occurrence. As a result of crashes, victims may be killed or slightly or severely injured. Passengers or pedestrians were also included in the analyses to capture more variability since the period of analysis is relatively short and they can indirectly cause collisions.

We used two outcomes: all alcohol-related crashes, and killed and seriously injured (KSI) alcohol-related victims. In both groups, drivers may be included. We used population information to determine rates in both outcomes. Population information was derived from the National Institute of Statistics [50]. We used this denominator instead of number of vehicles since the introduction of Uber could be associated with increments in this indicator, thus biasing the results.

To analyse the association of Uber with the outcomes, we considered two nine-month periods in both statistical analyses. The first period is between January 1st, 2013 and September 16th, 2013 (before the entry of Uber in Santiago). The second period starts on January 1st, 2014 with Uber’s entry and terminates on September 16th, 2014 with the enacted Emilia Law. This law imprisons drunk drivers for 1 year due to causing severe injury or death outcomes in a traffic crash [36]. We chose to limit the second period because other studies [51] have suggested that decreases of alcohol-related traffic crashes could be attributed to the Emilia Law, and therefore, estimating the true effect of ridesharing on the outcomes would become more problematic.

To cluster the 34 municipalities of Santiago according to five socioeconomic groups: High, High-middle, Middle, Middle-low, and Low, we used the methodology developed by the Observatory of Cities-UC [52]. This methodology uses individual survey data representative of each municipality from the National Institute of Statistics and government’s administrative data considering the following six dimensions: i) housing and neighbourhood (quality of housing construction, insecurity and street maintenance); ii) sociocultural conditions (social capital and education); iii) business development (conditions to invest, number of banks, unemployment, number of hotels); iv) work conditions (income, work contracts, debts, and life costs); v) health (access to health centres and exposure to air pollution); vi) and connectivity and mobility (access to public transport services and internet connection). In supplementary material, we described the socioeconomic classification per municipality.

### Spatial statistical analysis

Spatial autocorrelation is used to examine the level of spatial dependence between features according to their attribute values. Local spatial autocorrelation identifies spatial heterogeneity and distinguishes between spatial clusters of high- and low-value concentrations [53, 54]. The Getis-Ord Gi* statistic has been employed to perform local spatial autocorrelation analysis of road traffic crashes [55–60]. This statistic is expressed by Eq. (1), where *x*_*j*_ indicates the crash attribute at location *j*, *w*_*ij*_*(d)* is a spatial weight matrix for all locations *j* within a threshold distance *d* from the crash at location *i*, *n* is the total number of locations, and $\overline{x}$ and *S* are the mean and standard deviation, respectively.

1 $$ {G}_i^{\ast }(d)=\frac{\sum \limits_{j=1}^n{w}_{ij}(d){x}_j-\overline{x}\sum \limits_{j=1}^n{w}_{ij}(d)}{S\sqrt{\frac{n\sum \limits_{J=1}^n{w}_{ij}^2(d)-{\left(\sum \limits_{j=1}^n{w}_{ij}(d)\right)}^2}{n-1}}\ } $$

Positive and negative values for the Getis-Ord Gi* statistic represent clusters of crashes with high- and low-value events, respectively [61]. In this study, the Getis-Ord Gi* statistic was used to identify clusters of high values (hotspots) and clusters of low values (coldspots) of alcohol-related crash and KSI rates before and after the introduction of Uber in Santiago, Chile. Hotspots and coldspots were detected for three confidence levels (90, 95, and 99%).

### Risk ratios and random-effects meta-analysis

To complement our spatial statistical analysis, we also assessed alcohol-related crash variations by calculating risk ratios (RR) with 95% confidence intervals (CI) [62] at three levels: municipality, municipality socioeconomic groups, and Santiago. For this, we first determined the risk of experiencing an alcohol-related crash and KSI dividing each of these two outcomes by the population in each municipality. Second, to determine each RR, we divided the risk obtained after Uber’s entry by the risk before Uber’s entry. An RR equal to 1.0 indicates no difference risks among Uber’s pre- and post-periods, whereas an RR lower (higher) than 1.0 suggests a lower (higher) risk after the presence of Uber. Third, to obtain a RR for each municipal socioeconomic group and one for the whole city, we applied random-effects meta-analysis by pulling the RR obtained from each municipality [63]. Random-effects were chosen since it is assumed that Uber trips were not equal among municipality socioeconomic groups. To deal with zero cells, we applied the Peto method [64], which can provide robust pooled estimates when information of specific unit of analysis is absent [65]. This strategy is adequate to determine overall changes either at the city level or at each municipality socioeconomic group. We formally tested a possible gradient across municipality socioeconomic groups using meta-regression analysis [66]. All spatial and statistical analyses were performed using ArcGIS 10.5 and Stata 16 software, respectively.

## Results

In Table 1, we observe the distribution of both all alcohol-related crashes and KSI-alcohol-related population rates for the period of analysis. Before Uber, all alcohol-related crashes and KSI-alcohol-related rates per 100,000 population are equal to 9.08 and 1.63, respectively, whereas after the introduction of Uber both rates are equal to 10.23 and 1.65, respectively.

**Table 1:** All alcohol-related crashes, and killed and seriously injured alcohol-related victims per 100,000 population before (Jan-September 2013) and with (Jan-September 2014) Uber in Santiago, Chile

Outcomes	Period of analysis	
	Pre-Uber January 1st September 16th, 2013	With Uber January 1st September 16th, 2014
All alcohol-related crashes per 100,000 population	9.08	10.23
Killed and seriously injured alcohol-related victims per 100,000 population	1.63	1.65

**Fig. 1:**
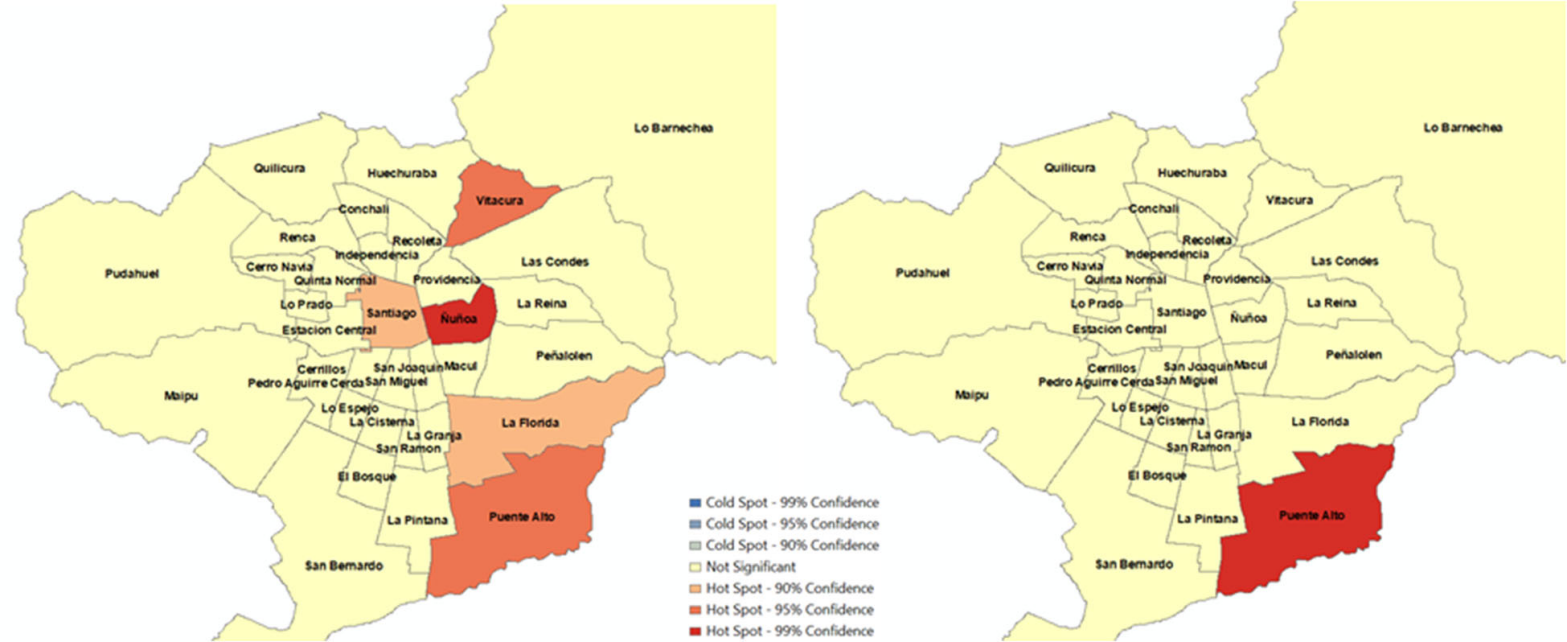
Hotspots of number of crashes in 34 municipalities, Santiago, Chile (January 1 to September 16, 2013 - January 1 to September 16, 2014). a) Before Uber’s entry. b) After Uber’s entry

**Fig. 2:**
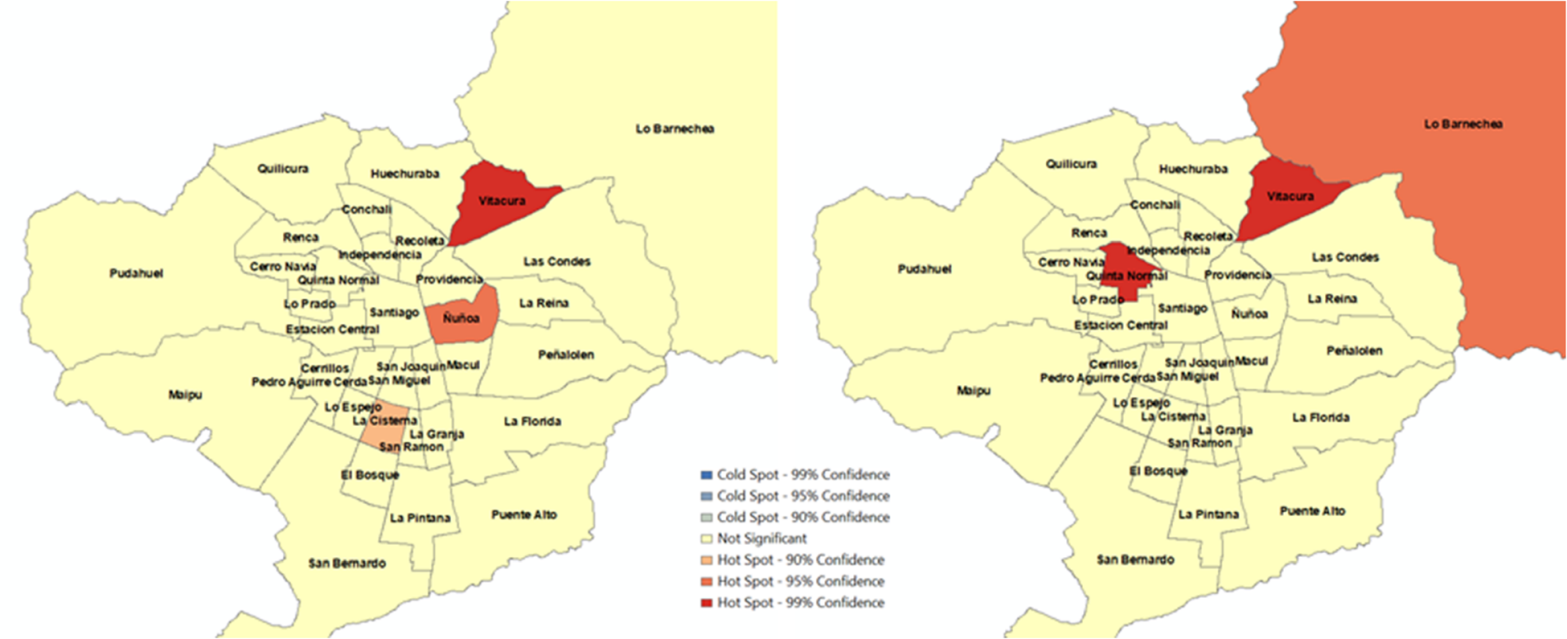
Hotspots of number of crashes per 100,000 population in 34 municipalities, Santiago, Chile (January 1 to September 16, 2013 - January 1 to September 16, 2014). a) Before Uber’s entry. b) After Uber’s entry

**Fig. 3:**
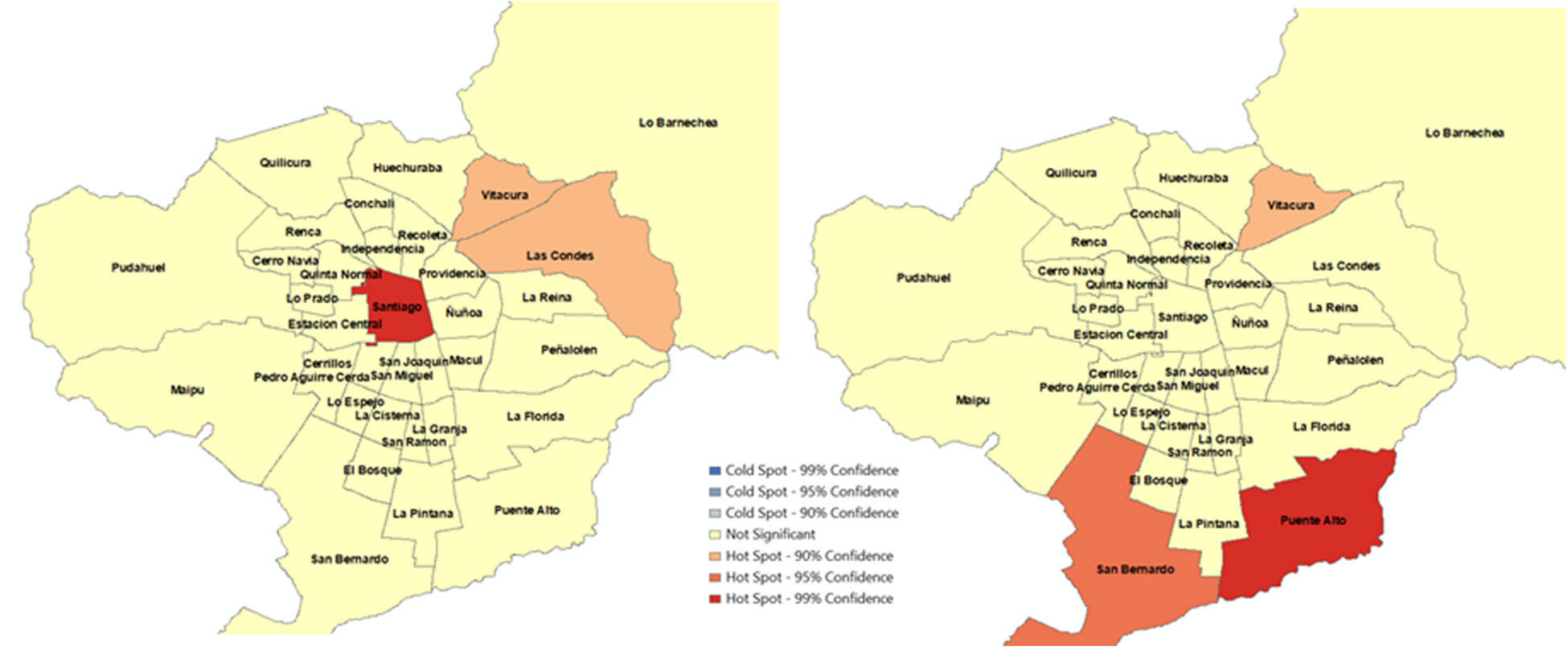
Hotspots of number of KSI in 34 municipalities, Santiago, Chile (January 1 to September 16, 2013 - January 1 to September 16, 2014). a) Before Uber’s entry. b) After Uber’s entry

**Fig. 4:**
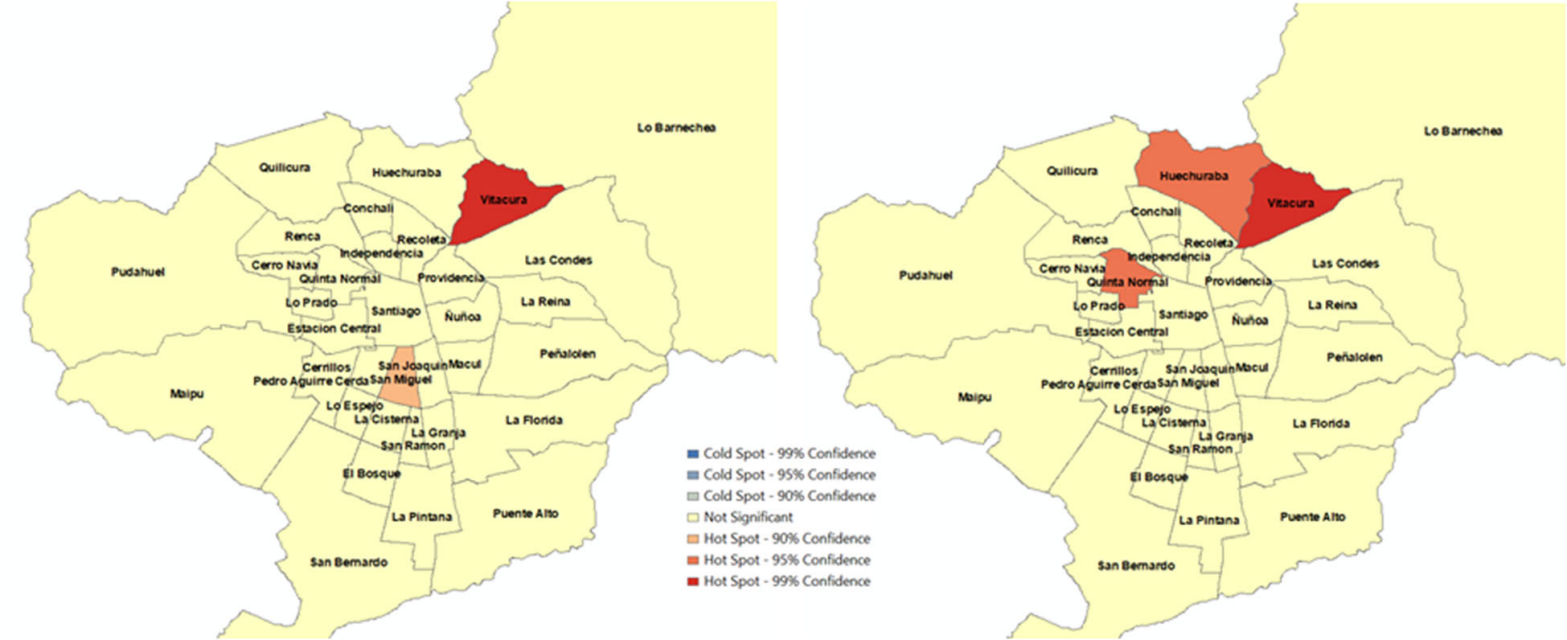
Hotspots of number of KSI per 100,000 population in 34 municipalities, Santiago, Chile (January 1 to September 16, 2013 - January 1 to September 16, 2014). a) Before Uber’s entry. b) After Uber’s entry

Figures 1-4 show spatial clustering of alcohol-related crashes and KSI rates during the period before and after the entry of Uber using the Getis-Ord Gi* statistic. Only hotspots (i.e., intense clustering of high values) are observed for different confidence levels in these figures, and thus, no coldspots (i.e., intense clustering of low values) were identified in the spatial statistical analysis. Figure 1 suggests that only the municipality of Puente Alto (middle SES) persisted as a hotspot of the total number of crashes with a 99% confidence level after Uber’s entry. Whereas Vitacura (high SES), Ñuñoa (High SES), Santiago (high-middle SES), and La Florida (high-middle SES) were no longer hotspots after the presence of Uber. Figure 2 suggests that Vitacura (high SES) persists over time as a hotspot when the analysis considers the number of crashes per 100,000 population. The municipalities of Ñuñoa (high SES) and La Cisterna (high-middle SES) were no longer hotspots in the period in which Uber emerges, but Quinta Normal (middle SES) and Lo Barnechea (high SES) arose as new hotspots in that period.

Hotspots for KSI outcomes were also mixed. Figure 3 suggests that Vitacura (high SES), Las Condes (high SES) and Santiago (high-middle SES) appeared as hotspots of KSI before Uber’s entry. In the period of Uber’s entry, only Vitacura (high SES) continued as a hotspot, but Santiago (high-middle SES) and Las Condes (high SES) were no longer hotspots. In the second period, San Bernardo (low-middle SES) and Puente Alto (middle SES) emerged as new hotspots. Figure 4 shows that Vitacura (high SES) persisted as a hotspot of the number of KSI per 100,000 population after Uber, but San Miguel (high-middle SES) was no longer a hotspot. The municipalities of Huechuraba (middle SES) and Quinta Normal (middle SES) emerged as new hotspots when Uber was launched in the city. Overall, these results do not show a consistent association between Uber’s pre- and post-periods and the observed alcohol-related crash variation. We obtained very similar results when we carried out analyses using vehicle fleet as denominator (Please refer to Figures SF1 and SF2).

**Fig. 5:**
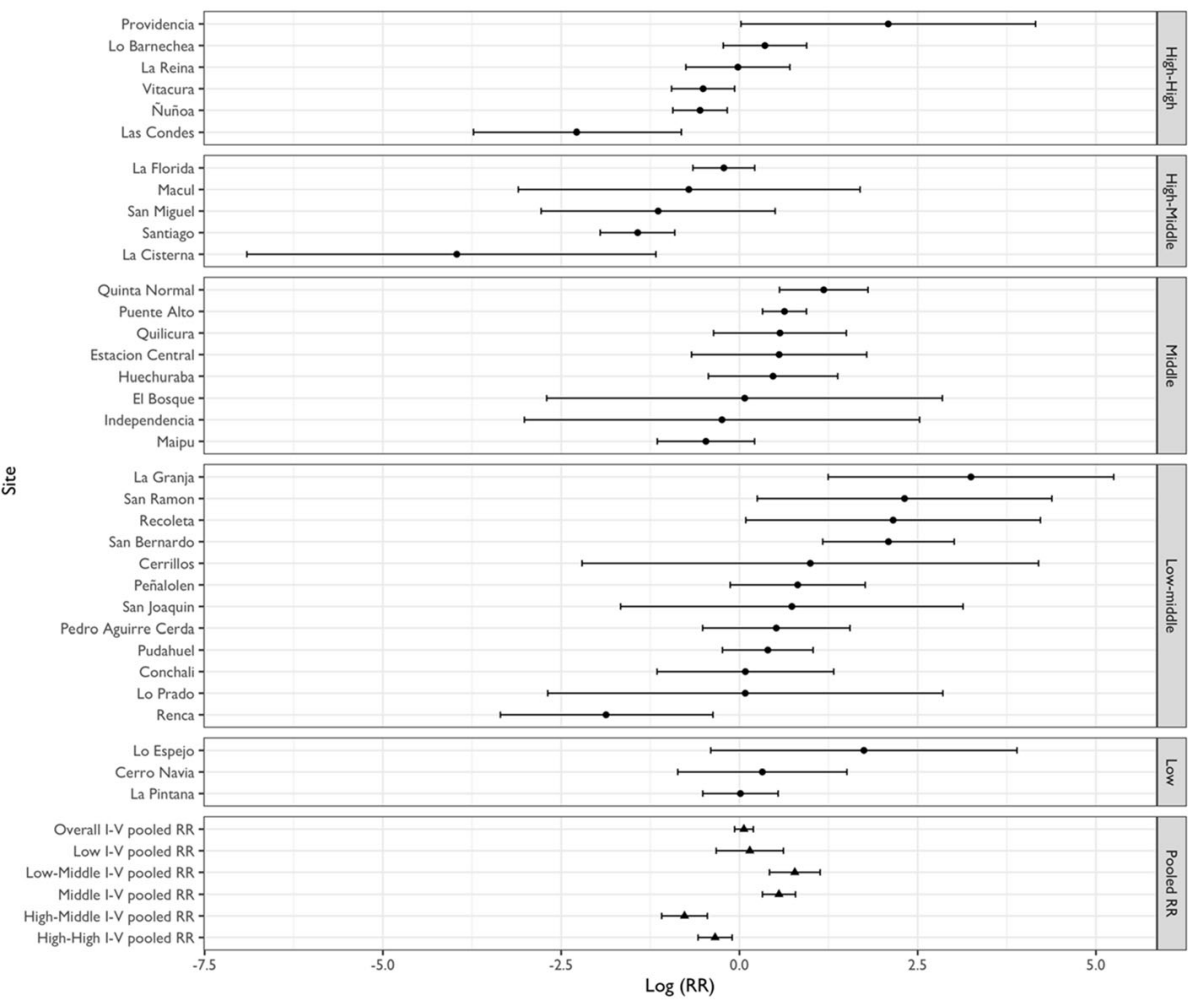
Association between alcohol-related crashes and absence and presence of Uber summarized by municipality socioeconomic groups

**Fig. 6:**
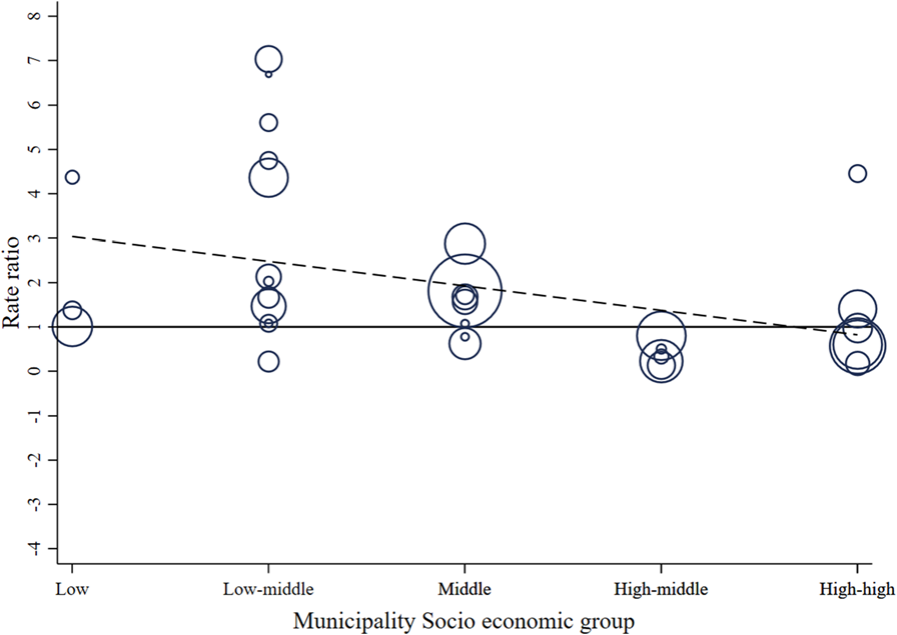
Bubble plot of alcohol-related crashes rate ratios with fitted meta regression line across municipality socio economic groups

**Fig. 7:**
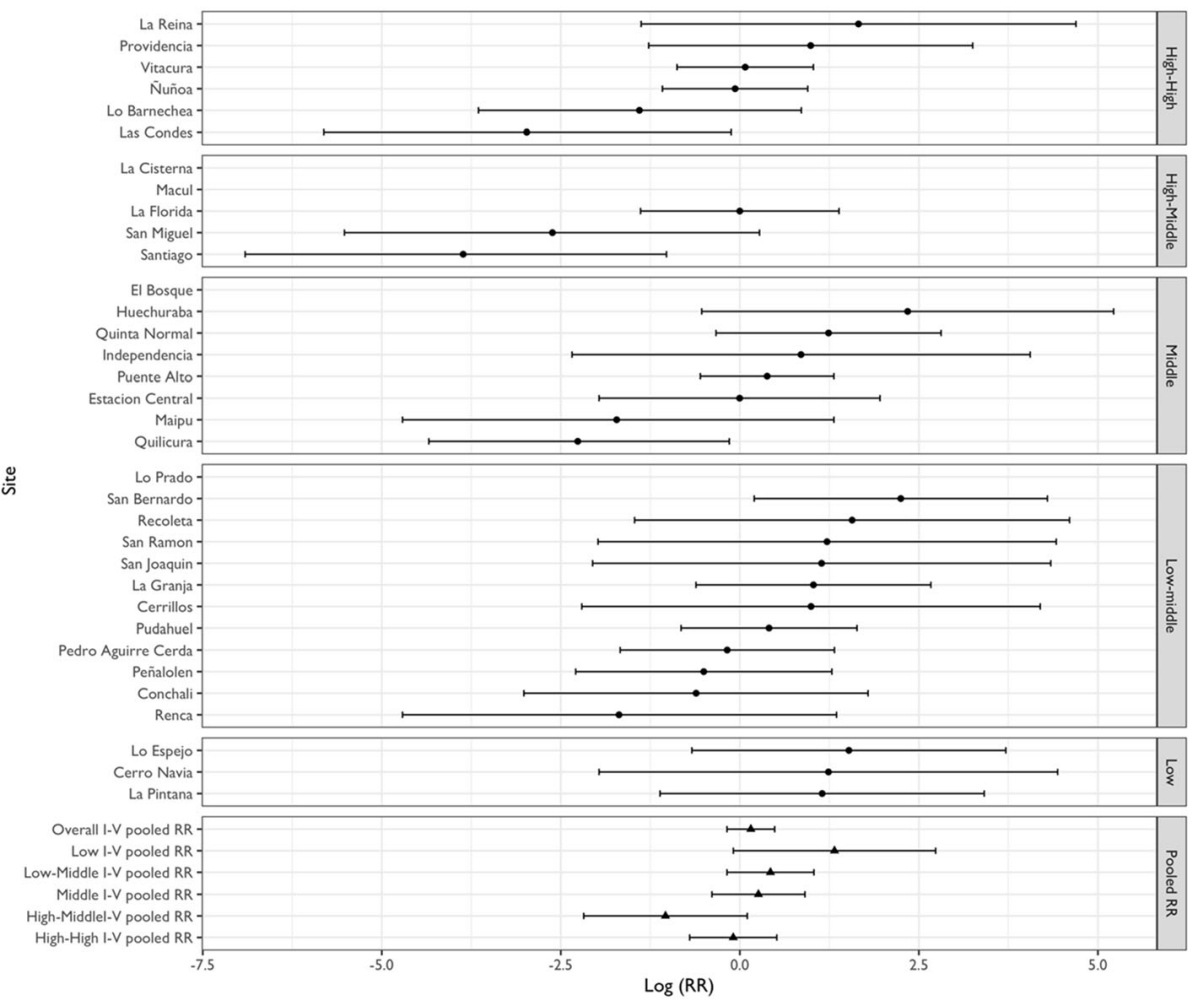
Association between KSI alcohol-related crashes and absence and presence of Uber summarized by municipality socioeconomic groups

**Fig. 8:**
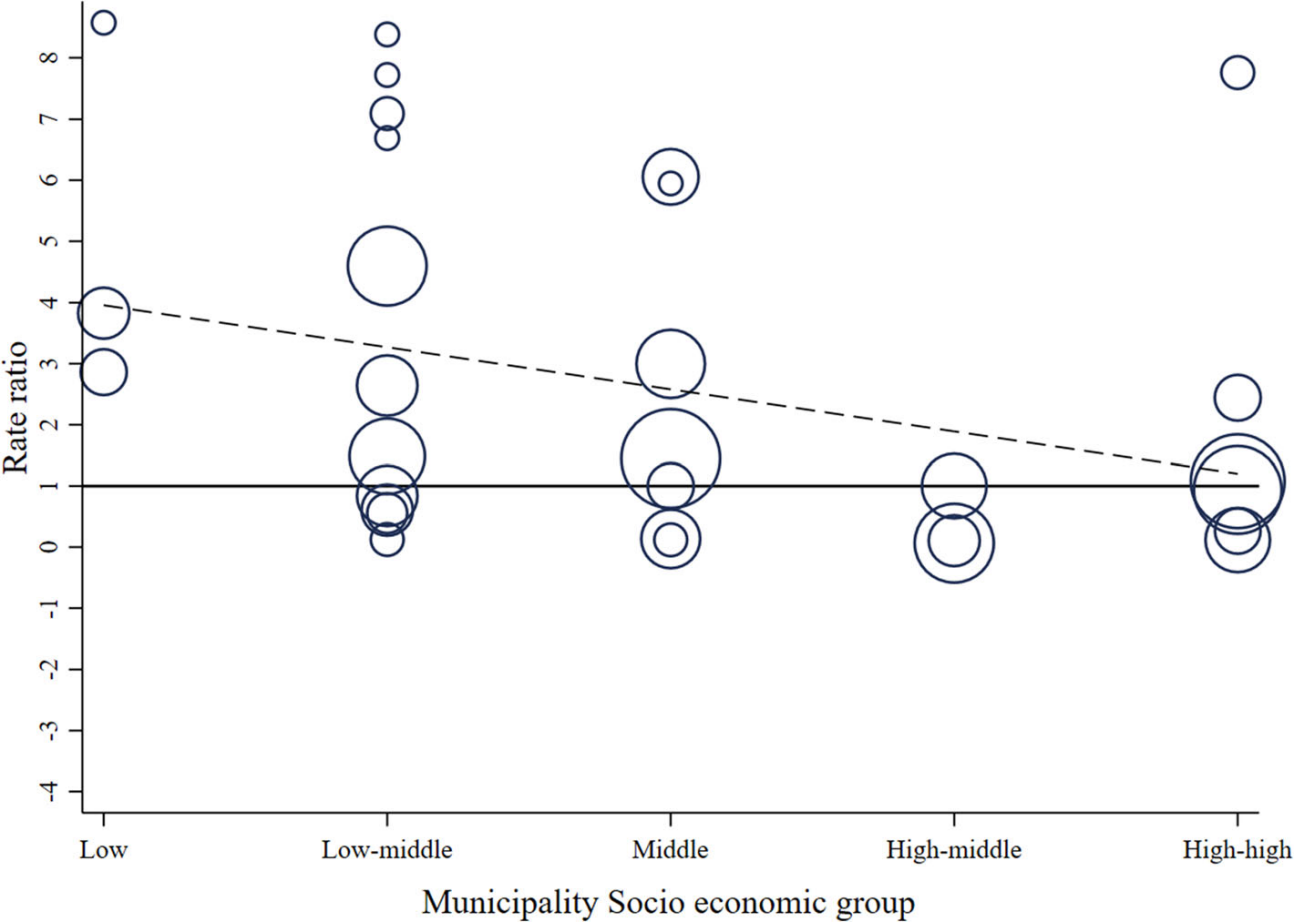
Bubble plot of alcohol-related crashes KSI rate ratios with fitted meta regression line across municipality socio economic groups

Figures 5 and 7 show RR for alcohol-related crash and KSI at the municipality level, pooled RR estimates by SES and the overall pooled RR estimate, respectively. Figures 6 and 8 display bubble plots of alcohol-related crashes RR and KSI, respectively, with fitted meta regression lines across municipality socioeconomic groups.

First, regarding alcohol-related crashes and Uber (Fig. 5), we observe a RR of 1.10 (95% C.I. 0.97, 1.27) for the overall association. This confirms the overall results of spatial analysis. Second, the high-high and high-middle groups are associated with RR of 0.71 (95% C.I. 0.56, 0.89) and 0.39 (95% C.I. 0.29, 0.52), respectively. The middle, low-middle, and low socioeconomic municipality groups have RRs higher than 1. Within the high-high group, Vitacura, Las Condes, and Ñuñoa have RRs of 0.61 (95% C.I. 0.38, 0.93), 0.18 (95% C.I. 0.07, 0.44) and 0.57 (95% C.I. 0.39, 0.84), respectively, and within the high-middle group, the municipalities of Santiago and La Cisterna have RRs in the same direction with 0.23 (95% C.I. 0.14, 0.38) and 0.14 (95% C.I. 0.06, 0.29), respectively. These results are partially aligned with the spatial analysis since similar tendencies were observed in Ñuñoa, Santiago, and La Cisterna. The clustering of RR by socioeconomic groups suggests a mild decrease gradient after the presence of Uber. The distribution of these results is captured in Fig. 6, in which the bubbles representing the RR of each municipality by SES show an inverse association after the introduction of Uber. The higher the SES of the municipality, the higher the reduction of alcohol related traffic outcomes.

Figure 7 confirms an overall no significant association in the city of Santiago between Uber and KSI variation as suggested in the spatial analysis. First, we observe a RR of 0.98 (95% C. I 0.73,1.31) for the overall estimate. Second, regarding socioeconomic groups, the high-high and high-middle groups are associated with RRs of 0.75 (95% C.I. 0.43, 1.31) and 0.17 (95% C.I. 0.08, 0.38), respectively, whereas middle, low-middle, and low groups have RRs higher than 1. Third, municipalities Las Condes, Santiago, and San Miguel have the lowest RRs with 0.12 (95% C.I. 0.03, 0.47), 0.06 (95% C.I. 0.02, 0.20) and 0.11 (95% C.I. 0.03, 0.47), respectively, and Huechuraba and San Bernardo are the only municipalities with positive and significant RRs of 6.06 (95% C.I. 1.21, 30.30) and 4.60 (95% C.I. 1.47, 14.30), respectively. Similarly, to alcohol-related crashes outcomes, there is a mild gradient across socioeconomic groups. Figure 8 confirms these observations, since the higher the socioeconomic classification of the municipality, the higher the reduction in the selected outcome. In short, the overall decline is pulled by municipalities classified as high and high-middle SES.

## Discussion

In Santiago, Chile, the first 260 days of ridesharing does not seem to be associated with variations on alcohol-related collisions per 100,000 population. However, this twofold analysis, considering 34 municipalities, suggests that in locations where residents with high SES live, the introduction of Uber could have had a mild protective effect, whereas in more vulnerable municipalities no effects and even increases in alcohol-related collisions were observed. The magnitude of prevention in municipalities, which disproportionally have the richest population, were associated with decreases of 29% in all alcohol-related collisions and a non-significant decrease of 25% in KSI outcomes. In the most disadvantageous zones, non-significant alcohol-related collisions increases of 20% but significant increases by three times in KSI were respectively observed.

The overall result of this study is in line with a previous study [36]. Nevertheless, the direction and magnitude of the present study is different. Whereas Nazif-Munoz et al. [36] found a non-significant decrease of 28% in alcohol-related collisions, in this study a non-significant 10% increase in the outcomes were observed. Differences across these two studies may be signalling variations between pre- and post-Uber terms used. Whereas the first study considered two and 1 years, respectively, in this one, the analysis was restricted to 9 months for each period. Results from the spatial analysis suggested that over time there are indeed differences across municipalities, which may have been masked when city is used as unit of analysis.

Assuming that ridesharing in its inception would penetrate high socioeconomic zones more rapidly, we hypothesized that Uber would have a protective effect in reducing alcohol-related outcomes in these areas. We specifically suggested that early adopters of ridesharing would have more opportunities to overcome the dilemma of drinking and driving since reaching an alternative means of private transport becomes more feasible when both a cell phone application and a credit card are available, and the ridesharing rates are affordable. This is reaffirmed when we concentrate on Las Condes and Vitacura. These two municipalities concentrate more than 63% of the individuals in the highest income quintile [67] and on average have the highest income per capita in Santiago [68]. Indeed, results of the spatial analysis suggests that Vitacura (Fig. 1) and Las Condes (Fig. 3) are no longer hotspots for the period when Uber emerges. Further, our RR suggest concomitant decreases by 29 and 82% in all alcohol-related collisions (Fig. 5), respectively. This socioeconomic gradient is indirectly confirmed with the associations observed across municipalities (Fig. 6). Possibly, alternative modes of transport such as ridesharing were not available because individuals living in these areas were structurally prevented to not having access to it since credit card was a required condition to use Uber.

The observed variability in the estimated relationships across municipalities before and after ridesharing was introduced may be however due to other circumstances, for instance, different levels of access to alcohol or alcohol consumption, police enforcement practices, and road infrastructure. Several studies conducted in Santiago [46, 69, 70] have suggested that income is not associated with increments in alcohol consumption. In fact, this has been noted using three different representative surveys, the Santiago Longitudinal Study, the National Health Survey 2009–2010, and the Family Budget Survey. As such, part of the decrease of alcohol-related collisions in high-income municipalities may not be attributed to decreases in alcohol consumption if no evidence so far points to this direction. However, increases in alcohol-related crashes in low socioeconomic municipalities could be associated with more access to unauthorized alcohol sales in these zones. Surveys in Chile suggest that perception of unauthorized alcohol sales in low socioeconomic municipalities have increased from 17.8% in 2012 to 23.7% in 2014 [71, 72]. In terms of police enforcement, spatial analysis of police stations within Santiago suggests a nonlinear socioeconomic distribution of access to police services [73]. Both lower and higher income groups are more likely to have less access to polices services than middle income groups. This may particularly explain the high RR of San Bernardo, but it is not adequate to understand why we observed over the period analysed a decrease in high-income municipalities and an increase in low-income municipalities. It is important to highlight that even though a no clear association across municipality socioeconomic groups and enforcement is observed in Santiago, there is also evidence that police enforcement within the same low socioeconomic groups may have very different practices, such as surveillance or detentions [74]. Whereas adequate police protection is consistently perceived in certain low socioeconomic neighbourhoods while in other zones this practice is not even present. This study suggests that low socioeconomic municipalities with high social capital have better communication with police officers, which in turn may affect the increment of policing in these territories [74]. Lastly, there are studies pointing that in Santiago in the last 10 years, transport infrastructure investment has indeed benefited individuals from the top quintiles [75–77]. Further, analyses considering the period 2000–2008 in Santiago suggested that the accumulation of specific crashes can be grouped within low quality infrastructure municipalities, however, these are not necessarily in the lowest income quintiles [76]. Changes in transport infrastructure could indeed be signalling decreases in KSI outcomes rather than all alcohol-related per se. One could assume that an increment of forgiving roads designed to decrease the severity of crashes in advantageous municipalities is likely a factor when explaining KSI differences across municipalities. However, this alternative explanation is not sufficient to understand all alcohol-related crashes because preventing alcohol behaviour is not a direct objective of infrastructure changes, but rather a function of enforcement, better transport alternatives, or alcohol availability [78].

There were several limitations to our approach. First, police assessment of alcohol involvement and classification of injuries and fatalities may not be consistent over time, and this measurement error might bias results. Second, it has been found that police officers, in Santiago, carry out identity controls more often to individuals from low socioeconomic groups than from high socioeconomic groups [79], and this practice can influence time to arrival to crash scenes biasing the results. Third, although we selected a period in which Uber operations were slowly increasing, we did not have continuous measures of rideshare utilization (e.g., counts of Uber journeys per municipality), much less whether the demand of this service varied by passenger’s income. These missing elements would help to better understand the relation between ridesharing and alcohol-related crashes. Last, we cannot rule out the potential for unmeasured time-varying confounding, and particularly the potential for other coincident externalities such as changes in gas prices, which affect overall mobility to impact rates of alcohol-related crash fatalities and injuries. In Santiago, there are considerable gas price differences across gas stations (for instance, more than 10% of difference can be found) [80], which affect the conditions of how individuals mobilize.

## Conclusions

This study highlights that to clearly understand the association between ridesharing (e.g., Uber) and alcohol-related crashes, attention to who its users are and where they live, and travel needs to be more carefully examined. This association, as such, is likely to differ across municipalities and over time depending on how many individuals increasingly adopt and use this technology vis-à-vis other existent transport alternatives when faced with the drinking and driving dilemma. Future studies should thus seek to identify the key aspects of these differential relationships considering characteristics of ridesharing users as well as the incorporation of other means of payment such direct cash. This study concludes that some technological changes can indeed be important platforms to protect populations exposed to negatives consequences associated with drinking and driving, but part of their success depends on how rapidly they expand. Nevertheless, more rigorous empirical studies should provide clearer explanations on how this novel technology becomes protective when passengers opt for it.

## Supplementary Information

**Additional file 1: Table S1.** Municipality by socioeconomic group classification. **Fig. S1.** Hotspots of number of crashes per 10,000 registered vehicles. **Fig. S2.** Hotspots of number of KSI per 10,000 registered vehicles.

## Data Availability

The datasets used and/or analysed during the current study available from the corresponding author on reasonable request.
