# Supplementary material for: Differential impacts of ridesharing on alcohol-related crashes by socioeconomic municipalities: rate of technology adoption matters
Source: BMC Public Health. 2021 Nov 4;21:2008. doi: 10.1186/s12889-021-12066-z (PMC8569979; doi:10.1186/s12889-021-12066-z)
Supplement: Supplementary file 1 — Additional file 1: Table S1. Municipality by socioeconomic group classification. Fig. S1. Hotspots of number of crashes per 10,000 registered vehicles. Fig. S2. Hotspots of number of KSI per 10,000 registered vehicles. [file 12889_2021_12066_MOESM1_ESM.docx]

**Supplementary material**

**Table S1 Municipality by socioeconomic group classification**

| **Municipality** | **Socioeconomic group classification** |
| --- | --- |
| Vitacura | High-High |
| Las Condes | High-High |
| Ñuñoa | High-High |
| Lo Barnechea | High-High |
| Providencia | High-High |
| La Reina | High-High |
| Santiago | High-Middle |
| San Miguel | High-Middle |
| La Florida | High-Middle |
| Macul | High-Middle |
| La Cisterna | High-Middle |
| Huechuraba | Middle |
| Quilicura | Middle |
| Independencia | Middle |
| Puente Alto | Middle |
| Maipu | Middle |
| Estación Central | Middle |
| El Bosque | Middle |
| Quinta Normal | Middle |
| Peñalolén | Low-middle |
| La Granja | Low-middle |
| San Ramon | Low-middle |
| Conchali | Low-middle |
| Renca | Low-middle |
| Pedro Aguirre Cerda | Low-middle |
| Pudahuel | Low-middle |
| Lo Prado | Low-middle |
| Cerrillos | Low-middle |
| San Bernardo | Low-middle |
| San Joaquín | Low-middle |
| Recoleta | Low-middle |
| La Pintana | Low |
| Lo Espejo | Low |
| Cerro Navia | Low |

**Figure S1**: Hotspots of number of crashes per 10,000 registered vehicles


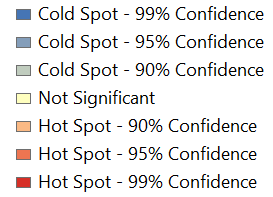

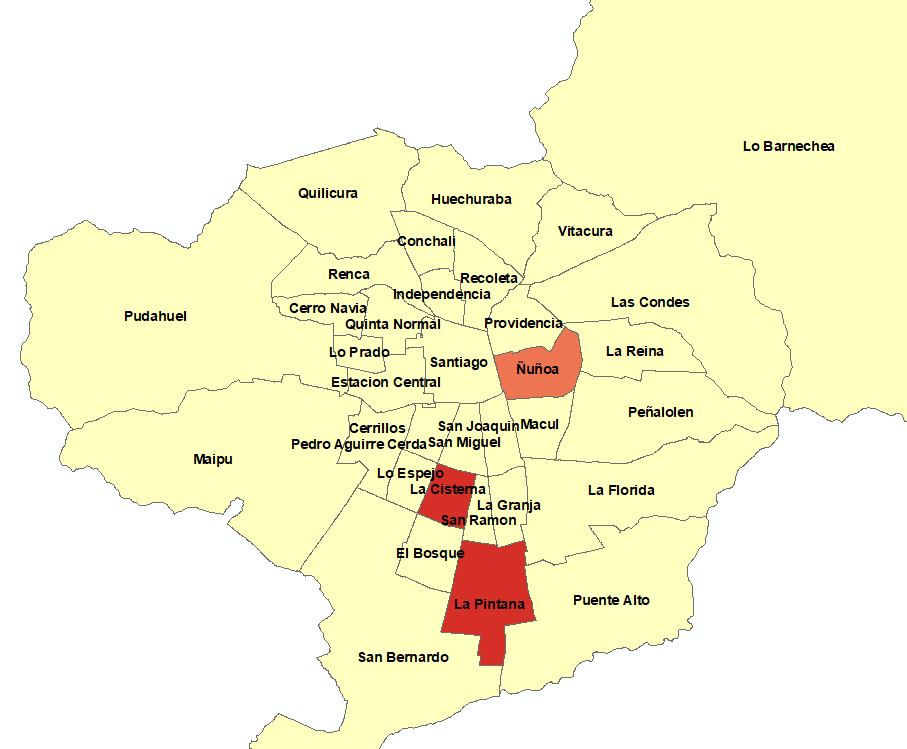

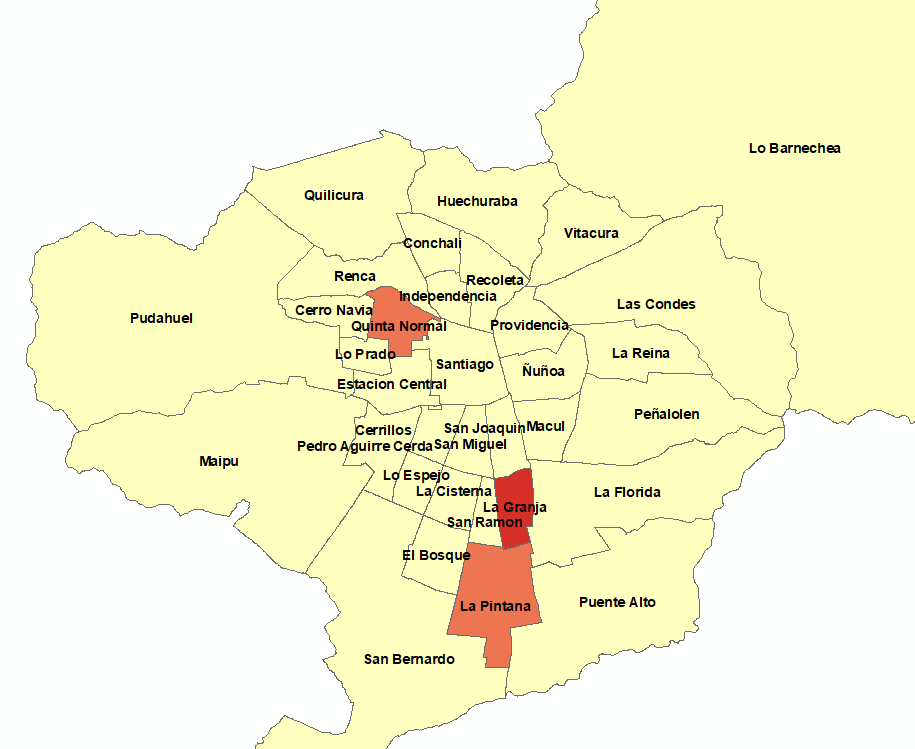


1. Before Uber´s entry b) After Uber´s entry

**Figure S2**: Hotspots of number of KSI per 10,000 registered vehicles


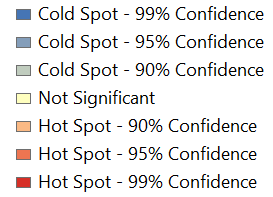

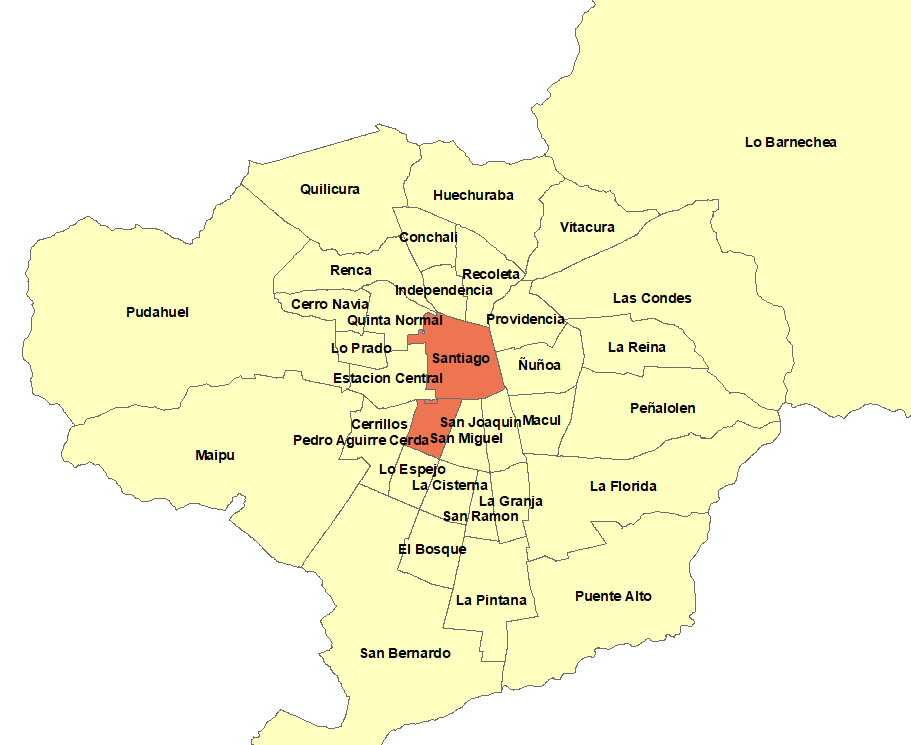

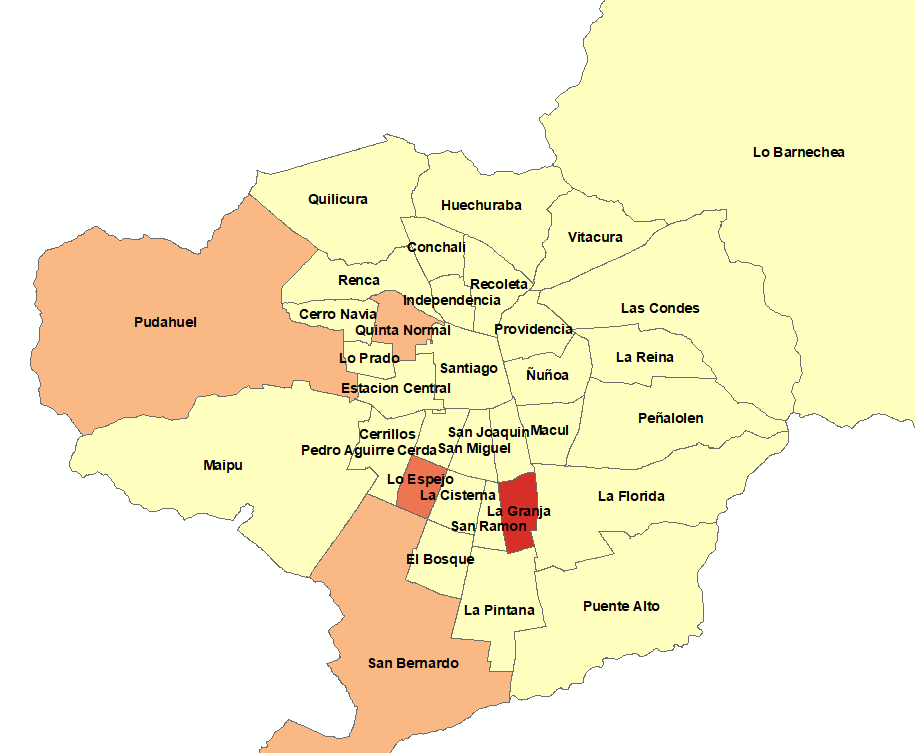


1. Before Uber´s entry b) After Uber´s entry
